# Transposition of the Viscera

**Published:** 1904-06-25

**Authors:** 


### TRANSPOSITION OF THE VISCERA.

Examples of complete or incomplete transposition of the viscera are reported from time to time and are generally regarded, probably correctly, as having little or no bearing on practice, however great may be their scientific interest. Even granting this, it is still very desirable that the description of these cases should be accurate and complete, and there is one respect in which these claims do not appear to be realised. This remark applies more particularly to the so-called transposed heart. What is wanted is not merely the statement that the cardiac impulse and dulness are found in the right chest, for this only proves displacement and not transposition in the usual acceptation of that term. Such facts are present in an ordinary case of dexiocardia due, for example, to a considerable left pleural effusion. But transposition implies more than this. It suggests not only that the heart lies mainly to the right instead of to the left of the middle line, but that its constituent parts have undergone a similar disturbance of their mutual relationship. The position is illustrated by what occurs in the case of the liver. Here it is obvious that transposition does not only mean that the organ lies mainly to the left instead of to the right of the middle line, but also that it is the left instead of the right lobe that has undergone the larger measure of development. Does a similar transformation occur in reference to the two sides of the heart, the right carrying on the systemic and the left the pulmonic circulation? It may be difficult to answer this question with confidence during life, but a well-taken skiagram might establish a strong presumption. Thus, amongst other facts it might show whether the aortic arch was directed towards the right or the left side. The importance of this observation does not seem to be generally appreciated, for the cases are usually put on record with a description which implies, it is true, a lateral displacement of the heart as a whole, but does not involve a corresponding change in the order of its constituent parts.

